# Laparoscopic Fluorescence Guided Detection of Uterine Niche—The Next Step in Surgical Diagnosis and Treatment

**DOI:** 10.3390/jcm11092657

**Published:** 2022-05-09

**Authors:** Harald Krentel, Lisa-Kathrin Lauterbach, Georgios Mavrogiannis, Rudy Leon De Wilde

**Affiliations:** 1 Department of Gynecology, Obstetrics and Reproductive Health, Peruvian University Cayetano Heredia, Lima 15000, Peru; 2 Clinic of Gynecology, Obstetrics, and Gynecological Oncology, Bethesda Hospital, 47053 Duisburg, Germany; gynklinik@bethesda.de (L.-K.L.); g.mavrogiannis@bethesda.de (G.M.); 3 Clinic of Gynecology, Obstetrics and Gynecological Oncology, University Hospital for Gynecology, Pius-Hospital Oldenburg, Medical Campus University of Oldenburg, 26121 Oldenburg, Germany; rudy-leon.dewilde@pius-hospital.de

**Keywords:** uterine niche, isthmocele, cesarean section, adenomyosis, dyspareunia, dysmenorrhea, ectopic pregnancy, laparoscopy, hysteroscopy

## Abstract

(1) Background: Uterine niche is a frequent condition in patients with a history of cesarean section. Although the relation to uterotomy seems to be clear, the exact pathogenesis is not fully understood. Uterine niche can easily be diagnosed by transvaginal ultrasound. It can be related to symptoms like dysmenorrhea, bleeding disorders, dysuria and dyspareunia. Uterine niche can be the cause of scar pregnancy, a rare form of ectopic pregnancy which can be related to severe complications; (2) Methods: We present a series of nine cases with different uterine niche related findings and discuss the diagnostic and therapeutic options reviewing the current literature and introduce a novel intrauterine ICG use for laparoscopic niche detection in one case; (3) Results: Most of uterine niche related symptoms and complications can be treated by a minimally invasive approach. Laparoscopic fluorescence guided niche detection is feasible; (4) Conclusions: Hysteroscopic and laparoscopic techniques allow the treatment of uterine niche related symptoms and complications. Intrauterine ICG application during fluorescence guided laparoscopy may allow easy niche detection.

## 1. Introduction

Uterine niche is a frequent finding in patients with a history of cesarean section [1]. It can easily be diagnosed during transvaginal ultrasound examination. The appearance of uterine niche can differ from dehiscence to cystic lesions. A classification of these different findings does not yet exist, and it remains unclear, if a uterine niche is a complication per se and, if treatment is generally necessary, especially in patients with a further wish to conceive. A uterine niche can cause symptoms like dysmenorrhea, dysuria, dyspareunia and bleeding disorders and spotting [2]. Uterine niche can be the reason for a niche or scar pregnancy, a rare type of ectopic pregnancy which can be related to severe complications such as loss of pregnancy, heavy uterine bleeding, uterine rupture, and loss of organs. In addition, in patients with intrauterine pregnancy, the presence of a uterine niche is concerning, although it is not yet clear if the presence of a niche is related to a higher rate of obstretrical problems like uterine rupture, premature birth, or placentation failure. The pathogenesis of uterine niche is not fully understood. The uterine suture technique during a cesarean section seems to be an important factor, but it remains unclear which technique is best in order to avoid this complication. Ectopic endometrial growth after uterine suturing also seems to play a role in the development of uterine niche and related symptoms. A standard for the treatment of uterine niche related problems does not exist. Niche related complications and symptoms can be treated by medical (hormonal) treatment or surgical approach. Hysteroscopy, laparoscopy and also open surgery are possible approaches depending on the type of niche and related complications. The laparoscopic resection of the uterine niche and scar and subsequent uterine suture seems to be the best surgical solution. Alternatively, hysteroscopic isthmoplasty is a minimally invasive treatment option. In the following, we present a series of cases and discuss diagnostic and therapeutic steps in patients with uterine niche related problems reviewing the current literature. As a novelty, we introduce a laparoscopic niche detection technique using intrauterine ICG application.

## 2. Materials and Methods

We retrospectively analyzed 9 cases with niche related symptoms and complications treated in our department of gynecological surgery during the period from 2019–2021 and introduce a new laparoscopic fluorescence guided niche detection technique. Table 1 shows patient age, the number of prior c-sections, the niche-related issues, the surgical treatment and the histopathological results. All patients have been treated by the principal author of this manuscript and all patients gave full written informed consent. All patients underwent 2D transvaginal ultrasound examination with niche detection and evaluation niche pregnancies, size of niche and residual myometrial thickness (RMT). The surgeries have been carried out using the standard surgical set-up of our department including diagnostic hysteroscopy, bipolar hysteroresectoscopy (Karl Storz SE & Co. KG, Tuttlingen, Germany) and 4K-laparoscopy (Arthrex GmbH, München, Germany) with the option of fluorescence-guided laparoscopy. We review the pubmed database using the keywords uterine niche, isthmocele, cesarean section, adenomyosis, dyspareunia, dysmenorrhea, ectopic pregnancy, laparoscopy and hysteroscopy.

## 3. Results

From 2019 to 2021, we identified nine cases of uterine niche and isthmocele related to different complications and symptoms. The increasing number of laparoscopic surgeries in niche-related conditions led us to the development of a new surgical niche detection technique using fluorescence guided laparoscopy (Case 1). In five cases, the patients presented with a niche pregnancy (Cases 2–6). One of these patients presented with a uterine rupture, acute abdomen and severe uterine bleeding. Due to this life-threatening event, we performed emergency hysterectomy in this case (Case 3). One patient with adenomyosis related symptoms and niche pregnancy opted for a definitive solution, and we performed a laparoscopic subtotal hysterectomy (Case 4). In two patients with niche pregnancy, we performed bipolar hysteroscopic resection and subsequent laparoscopic niche repair (Cases 2 and 6), while in one patient we directly resected the niche including the early pregnancy by laparoscopy (Case 5). One patient presented with a large symptomatic cystic uterine niche or isthmocele (Case 9). Three patients (Cases 1, 7 and 8) presented with a symptomatic uterine niche. Symptoms included bleeding disorders and spotting, dysuria, dysmenorrhea and dyspareunia. Histopathology revealed adenomyosis within the resected uterine scar in these three cases. In 5/9 patients, adenomyosis was found in the resected uterine niche.

### 3.1. Case 1—Symptomatic Niche—Laparoscopic Treatment with ICG Niche Detection

**Figure 1:**
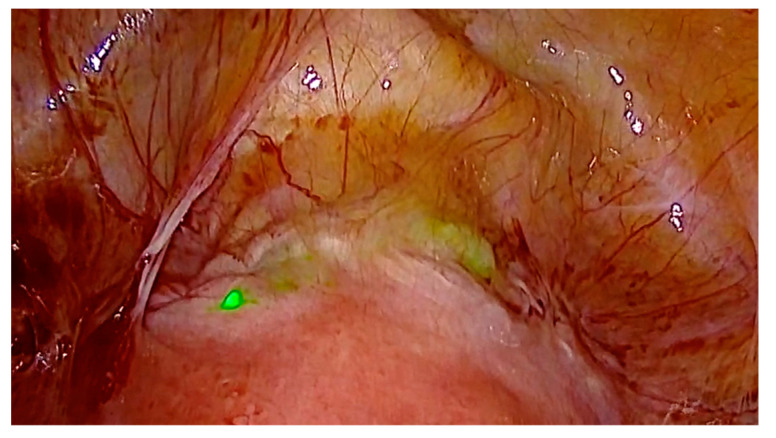
Laparoscopic fluorescence-guided view of uterine niche after intrauterine application of indocyanine green solution.

This 34-year-old patient presented with bleeding disorders including hypermenorrhea and irregular spotting, dysuria, dysmenorrhea and dyspareunia. Transvaginal ultrasound examination revealed a uterine niche and adenomyosis. After informed consent considering medical treatment options, we performed a laparoscopic subtotal hysterectomy with resection of the uterine niche. In order to easily detect the uterine scar, we used an indocyanin green solution (diagnostic Green GmbH, Aschheim-Dornach, Germany) and a laparoscopic fluorescence technique (Figure 1). We inserted a uterine manipulator (Rumi II, Cooper Surgical, Trumbull, CT, USA) and, after establishing a laparoscopic view, we applied 20 mL of ICG solution (5 mg in 100 mL Aqua) to the uterine cavity. The uterine niche was immediately visible in the anterior isthmocervical area by using a 4K-ICG-fluorescence technique (Arthrex GmbH, München, Germany). Thus, we easily determined the resection line and avoided unnecessary adhesiolysis and tissue preparation. The histopathological examination revealed adenomyosis in the uterine scar and the uterine corpus. In this case, any additional endometriosis was found (#Enzian FA).

### 3.2. Case 2—Niche Pregnancy—Combined Hysteroscopic and Laparoscopic Treatment

**Figure 2:**
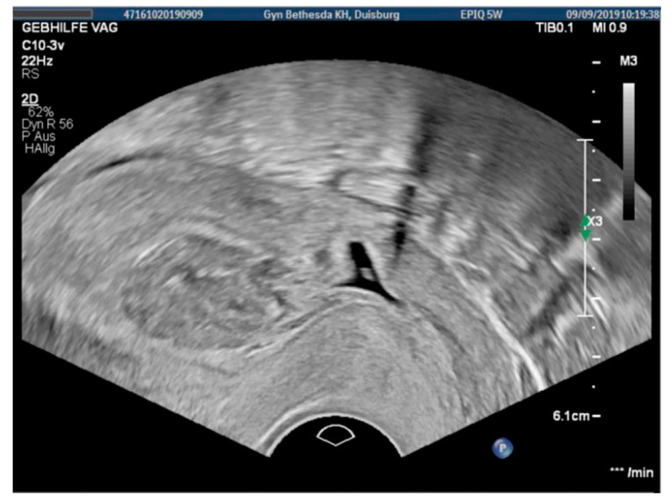
Transvaginal ultrasound showing a niche pregnancy with 6 weeks of gestation.

The 31-year-old patient presented with an asymptomatic uterine niche pregnancy in the 7th week of gestation as a result of in vitro fertilization. Before, the patient underwent two cesarean sections, laparoscopic adhesiolysis, and appendectomy. Transvaginal ultrasound revealed an early niche pregnancy (Figure 2) and the blood samples showed a positive hCG (presurgical: 14614 IU/l). We performed the hysteroscopic resection of the pregnancy using a bipolar hysteroresectoscope and an abrasion of the uterine cavity without complications. The blood loss during this procedure was minimal. The postsurgical hemoglobin was 13.3 g/dL. In a subsequent laparoscopic surgery 9 weeks later, we resected the uterine scar and sutured the uterus with a 2-0 PDS barbed suture. The histoptahological examination of the uterine scar revealed adenomyosis and no remains of the chorionic villi. Additionally, we found a mild peritoneal endometriosis (#Enzian P1 FA) in this patient. The postsurgical hemoglobin was 12.3 g/dL.

### 3.3. Case 3—Niche Placentation Failure—Emergency Hysterectomy

The 40-year-old patient presented with acute abdominal pain with 13 weeks of gestation in a reduced general status. She reported a central pelvic pain for two weeks. In the past, she had a vaginal delivery in 2003 and a cesarean section due to placenta praevia in 2013. Before this pregnancy, she underwent assisted reproduction. Clinical examination revealed the signs of an acute abdomen. The ultrasound examination showed a hemoperitoneum and an intrauterine fetus with positive cardial function. We decided to perform an emergency laparoscopy which revealed a uterine rupture and severe uterine bleeding with abruption of the placenta in the site of the uterine scar. Due to the severity of the bleeding, we converted to laparotomy and finally decided to perform an emergency hysterectomy in order to save the patient’s life. The preservation of the fetus and reconstruction of the uterus were no options in this life-threatening situation. The presurgical hemoglobin was 10.1 g/dL. During and after the surgery, the patient received several blood transfusions and left the hospital with a hemoglobin of 8.0 g/dL. The histopathological examination showed a uterine rupture in the area of the c-section scar. They reported a placentation failure with a placenta praevia and increta in the anterior isthmocervical region. Additionally, several uterine fibroids were found but no adenomyosis.

### 3.4. Case 4—Niche Pregnancy—Laparoscopic Subtotal Hysterectomy

The 39-year-old patient presented with symptomatic adenomyosis and moderate vaginal bleeding. Transvaginal ultrasound revealed uterine niche pregnancy with 8 weeks of gestation and adenomyosis of the myometrium of the anterior and posterior uterine wall. After explaining the medical and surgical treatment options, the patient opted for a subtotal laparoscopic hysterectomy including the resection of the uterine niche and niche pregnancy. We performed this surgery with laparoscopic in-bag morcellation. The tissue of the uterine scar was sent separately to pathological examination. Adenomyosis was found in both uterine corpus and scar. No additional endometriosis was found during laparoscopy (#Enzian FA). The blood loss during surgery was minimal.

### 3.5. Case 5—Niche Pregnancy—Laparoscopic Resection and Repair

**Figure 3:**
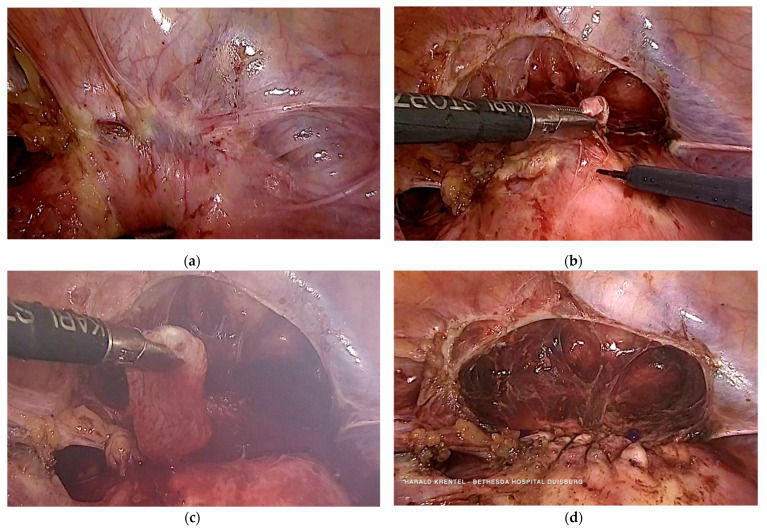
Laparoscopic treatment of niche pregnancy (7 weeks of gestation). (**a**) laparoscopic view of the uterine niche pregnancy; (**b**) laparoscopic resection of the uterine scar and niche pregnancy using a monopolar needle; (**c**) laparoscopic removal of the gestational sac; (**d**) laparoscopic view after double layer suture of the uterine wall.

The 40-year-old patient presented with moderate vaginal bleeding and the suspected diagnosis of missed abortion with seven weeks of gestation. Before, she underwent three cesarean sections and two vaginal deliveries. She experienced preeclampsia and once a deep vein thrombosis two times. Transvaginal ultrasound revealed a uterine scar pregnancy. We indicated and performed a laparoscopic resection of the uterine niche including the pregnancy and sutured the uterus with a double-layer using barbed suture (Figure 3). The blood loss during surgery was 250 mL. The postsurgical hemoglobin was 11.8 g/dL. Histopathology reported early pregnancy and decidualised endometrium surrounded by fibrotic tissue with signs of chronic inflammation.

### 3.6. Case 6—Niche Pregnancy—Combined Hysteroscopic and Laparoscopic Treatment

The 43-year-old patient presented with vaginal bleeding due to uterine niche pregnancy in the 9th week of gestation. After transvaginal ultrasound diagnosis, we performed a bipolar hysteroresectoscopy with complete resection of the pregnancy related tissue without any complications. Histopathological examination revealed chorionic villi and decidua. The postsurgical hemoglobin was 12.3 g/dL. Four days after surgery, the pregnancy test was negative. Three months after the hysteroscopic resection of the niche pregnancy, we performed a laparoscopic resection of the uterine niche with laparoscopic uterine closure using a barbed PDS suture. During histopathological examination, no adenomyosis of the uterine scar and no remnants of the chorionic villi were found. There was no peritoneal endometriosis in this patient.

### 3.7. Case 7—Symptomatic Niche—Laparoscopic Treatment

**Figure 4:**
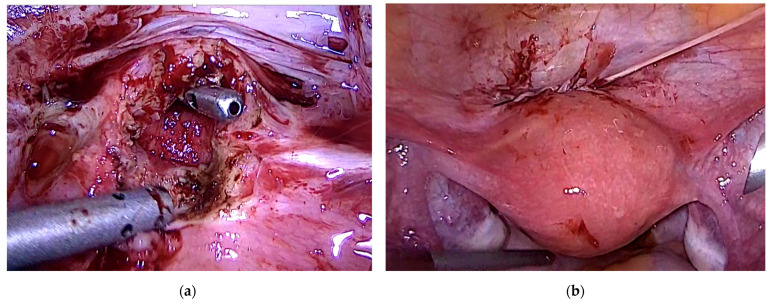
Laparoscopic treatment of symptomatic uterine niche pregnancy. (**a**) laparoscopic view of the uterine cervix after niche resection; (**b**) laparoscopic view after double layer suture of the uterine wall and the peritoneum.

The 27-year-old patient presented with pelvic pain and dysmenorrhea and a history of a cesarean section. Transvaginal ultrasound examination showed a uterine niche with suspicion of local adenomyosis. We performed diagnostic hysteroscopy and laparoscopic resection of the uterine niche with double-layer barbed suture (Figure 4). Histopathology revealed adenomyosis with the scar. An additional endometriosis was found during surgery (#Enzian P2 FA).

### 3.8. Case 8—Symptomatic Niche—Laparoscopic Treatment

**Figure 5:**
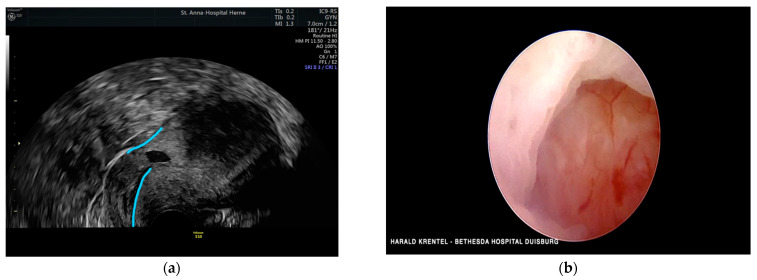
(**a**) Transvaginal ultrasound showing a uterine niche; (**b**) hysteroscopic view of uterine niche.

The 31-year-old patient presented with dysmenorrhea and bleeding disorders including spotting. Transvaginal ultrasound examination revealed a uterine niche with RMT < 3 mm. We performed diagnostic hysteroscopy and laparoscopic resection of uterine niche and laparoscopic resection of peritoneal and deep endometriosis. We closed the uterus with a double-layer barbed PDS suture. The histopathological examination revealed adenomyosis of the uterine scar and an additional peritoneal and deep endometriosis (#Enzian P1 B1/0 FA). The blood loss during surgery was minimal (Figure 5).

### 3.9. Case 9 —Symptomatic Isthmozele—Laparoscopic Treatment

**Figure 6:**
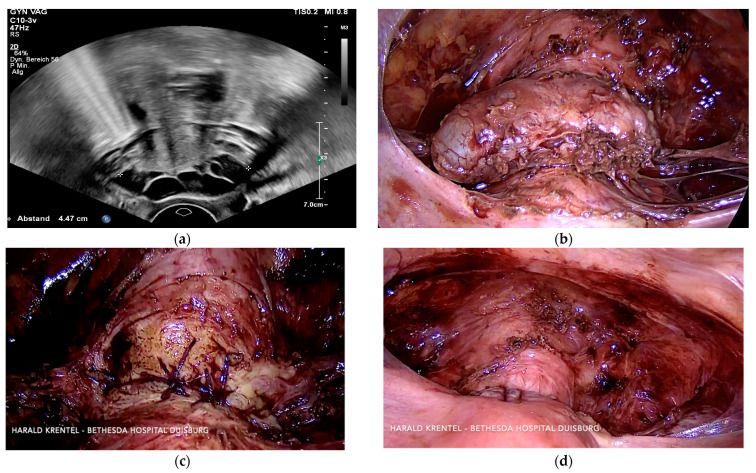
(**a**) Transversal transvaginal ultrasound showing a large isthmocele in the anterior uterine wall; (**b**) laparoscopic view of isthmocele after deperitonealisation and detachment of bladder; (**c**) laparoscopic suture—first layer; (**d**) laparoscopic suture—second layer.

**Figure 7:**
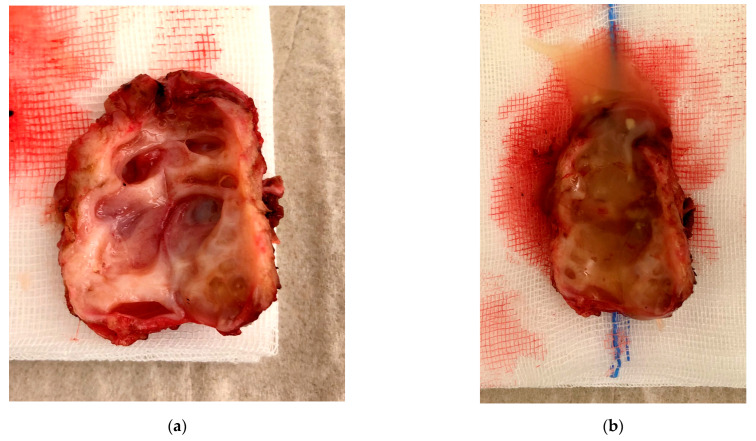
Specimen after resection. (**a**) inner structure of isthmocele; (**b**) isthmocele with mucus.

This 33-year-old patient presented with a symptomatic cystic lesion in the anterior uterine wall after cesarean section. Transvaginal ultrasound showed a large cystic isthmocele with a maximum diameter of 4.47 cm. We performed laparoscopic resection and a double-layer suture with a barbed PDS thread. The blood loss during surgery was minimal (Figure 6 and Figure 7).

## 4. Discussion

### 4.1. Niche Detection with Fluorescence Guided Laparoscopy

Uterine niche represents an iatrogenic myometrial defect of the isthmocervical anterior uterine wall at the site of caesarean scar due to defective tissue healing. Uterine niche is a gap in the uterine wall mostly covered by a thin layer of myometrium or peritoneum corresponding to the uterine cavity. The niche can be linear, bell-mouthed, pyramidal, hypoechoic or hyperechoic. Uterine niche and isthmocele can be found after one or more cesarean sections. The surgical management of niche, isthmocele, uteroperitoneal fistula or cesarean scar defect has been described by Nezhat et al., and Jacobson et al. [3,4]. The detection of uterine niche during laparoscopy can be challenging. After cesarian section, there can be adhesions between bladder and anterior uterine wall which are covering the uterine scar. In order to detect the uterine niche, different authors describe a combined hysteroscopic and laparoscopic approach [5]. This light-guided procedure requires two independent optical systems during surgery. We present a novel approach in laparoscopic detection of a uterine niche by using fluorescence-guided surgery with indocyanin green (ICG) application (Figure 1). After the positioning and desinfection of the patient, a uterine manipulator is attached to the uterus (RUMI II, Cooper Surgical, Trumbull, CT, USA). Under laparoscopic fluorescence-guided vision (Arthrex GmbH, München, Germany) of the anterior uterine region, a diluted ICG-solution is applied to the uterine cavity. Immediately after ICG-solution application, the fluorescence signal clearly shows the uterine scar, respectively, niche. This way, the uterine niche can be easily detected without a second optical system, and without complex tissue preparation. The resection of the uterine scar can be performed targeted. To our knowledge, this is the first report of this novel method, which helps to avoid unnecessary tissue preparation and may help to minimize the risk of bladder lesions during niche resection.

### 4.2. Uterine Niche—Is a Treatment Necessary?

Di Spiezio Sardo et al., analyzed the risk of uterine scar defects in relation to the suturing technique during cesarian section including nine RCTs with a total of 3969 patients. Interestingly, the risk of uterine scar defects and respective complications is similar in patients with a single-layer suture compared to a double-layer suture [6]. Marchand et al., showed that a double-layer suture results in a higher residual myometrial thickness, but not in a lower rate of isthmocele [7]. De Luget et al., recently discussed the possible risk factors for uterine niche, including but not limited to the timing of cesarean section, location of incision, techniques for opening and closing of the uterine wall and the bladder detachment. The most important factor in order to reduce the risk of uterine niche related complications is a reduction of the rate of the first caesarean section, followed by a rather high incision at a distance from the internal os [8]. Based on a systematic review of the literature including 31 articles, Mashiach and Burke recently reported that patients with a cesarean scar defect are usually asymptomatic. In case of symptoms, the surgical repair by hysteroscopy or laparoscopy is considered as a valid option. For patients with a residual myometrial thickness (RMT) of >2–3 mm, the hysterocopic approach seems suitable, whereas the laparoscopic repair should be used in RMT < 2.5 mm [5]. Accordingly, Tanos et al., reported that a surgical repair of uterine niche can be performed when the residual myometrial thickness is below 3 mm [9]. Di Spiezio Sardo et al., described the minimally invasive technique of hysteroscopic isthmoplasty as a treatment option to improve postmenstrual bleeding in patients with an RMT of at least 3 mm. In an RMT less than 3 mm, the risk of bladder injury can increase [10]. The efficacy and benefits of laparoscopic isthmocele repair have been recently shown by Karampelas et al. In 31 patients with cesarean scar defect, an increase of the RMT from 1.77 mm to 6.67 mm postoperatively in combination with improvement of uterine bleeding disorders, chronic pelvic pain, and secondary infertility has been described [11]. The new fluorescence guided technique might ease the minimally invasive niche detection and repair in such cases.

### 4.3. Uterine Niche and Adenomyosis

AbdulGaffar et al., described the histopathological findings in 22 cases of hysteroscopy-resected isthmoceles. The resected isthmocele edges were lined by endocervical, endometrial, and isthmic mucosa either combined or isolated depending on each case [12]. Karpathiou et al., described endocervical mucosa as the most frequent histopathologic feature in uterine niche, combined with regenerative epithelial atypia and fibroblastic stromal reaction. These findings cannot be found in caesarean section scars without niche formation [13]. In our retrospective analysis, in 5/9 cases with uterine scar related symptoms and complications, adenomyosis has been found in the histopathological examination of the uterine scar. Symptomatic niche including bleeding disorders, brownish spotting, dysmenorrhea, dyspareunia and dysuria were related to the presence of adenomyosis within the resected uterine niche.

### 4.4. Uterine Niche Pregnancy

Uterine scar pregnancy represents a rare complication and can be related to severe complications such as uterine rupture, heavy bleeding, blood transfusion and organ loss. Uterine scar pregnancy (CSP) is differentiated into two types. Type I with progression to the uterine cavity or cervicoisthmic space and type II with progression to the bladder and the abdominal cavity. An evidence-based treatment approach does not exist. In a systematic review, Birch Petersen et al., concluded that surgical treatment by laparoscopy, hysteroscopy, vaginal approach and uterine artery embolization (UAE) in combination with dilatation and curettage is recommendable rather than a medical approach [14]. Maheux-Lacroix showed that laparoscopic, vaginal and open resection and repair are associated with a high success rate and a low risk of hemorrhage. Hysteroscopic resection was unsuccessful in 12% of cases and thus related to subsequent interventions. Dilatation and curettage alone are related to a high risk of hemorrhage but can be recommended in combination with UAE [15]. Treatment with local or systemic methotrexate (MTX) alone is not convincing, but some authors describe a combined medical and surgical approach with presurgical MTX treatment with good results [16]. Gestational age, maximum transverse diameter of gestational sac and myometrial residual thickness have been identified as factors in favor of laparoscopic resection and repair [17]. Women with a prior cesarean scar pregnancy have a high risk of recurrence, abortion, preterm birth and placentation failure [18]. In our retrospective analysis, laparoscopic and hysteroscopic treatment showed good clinical results without any need for reintervention due to residual pregnancy tissue. In two cases, we opted for a secondary laparoscopic niche repair after a primary hysteroscopic approach, as both patients wished to conceive in the future. Whether laparoscopic fluorescence guided niche detection is also feasible in niche pregnancy and cystic isthmocele and whether there is a relation to the RMT or the size of the gestational sac or cystic lesion have to be shown in further investigations.

## 5. Conclusions

Surgical treatment of niche related symptoms and complications by hysteroscopy and laparoscopy is safe and effective. Laparoscopic fluorescence-guided niche detection represents a novel approach which might help to prevent bladder lesions and unnecessary tissue preparation.

## Figures and Tables

**Table 1:** Basic data of 9 patients with uterine niche related symptoms and complications.

	Age	Number of C-Sections	Niche Related Issue	Treatment	Histopathology of the Niche
Case 1	34	2	Bleeding disorder, dysuria, dyspareunia	Laparoscopic ICG niche detection and resection	Adenomyosis
Case 2	31	2	Niche pregnancy, 6 weeks after IVF Pelvic pain and vaginal bleeding	Hysteroscopic resection, second look laparoscopy with scar resection and suture	Chorionic villi, decidua, adenomyosis
Case 3	40	1	Niche pregnancy with placenta increta and uterine rupture, 13 weeks	Emergency hysterectomy	Placenta praevia, Placenta increta, uterine fibroids, no adenomyosis
Case 4	39	2	Niche pregnancy, 8 weeks, dysmenorrhea, vaginal bleeding	Subtotal hysterectomy with scar resection (patients wish)	Chorionic villi, decidua, adenomyosis
Case 5	40	3	Niche pregnancy, 7 weeks of gestation	Laparoscopic resection and repair	Chorionic villi, decidua, no adenomyosis
Case 6	43	1	Niche pregnancy, 9 weeks, IVF	Hysteroscopic resection second look laparoscopy with resection and repair	Chorionic villi, decidua, no adenomyosis
Case 7	27	1	Dysmenorrhea and pelvic pain, subfertility	Laparoscopic niche resection and suturing	Adenomyosis
Case 8	31	1	Bleeding disorder, dysmenorrhea	Laparoscopic niche repair	Adenomyosis
Case 9	36	2	Large symptomatic isthmocele	Laparoscopic resection and repair	No adenomyosis

## Data Availability

The clinical data used to support the findings of this study are stored at Department of Gynecology, Obstetrics, Gynecological Oncology and Senology, Academic Teaching Hospital, Bethesda Hospital, Duisburg, Germany and are available from the corresponding author upon request.

## References

[1] Kulshrestha V., Agarwal N., Kachhawa G. (2020). Post-Caesarean Niche (Isthmocele) in Uterine Scar: An Update. J. Obstet. Gynaecol. India.

[2] Bij de Vaate A.J., van der Voet L.F., Naji O., Witmer M., Veersema S., Brölmann H.A., Bourne T., Huirne J.A. (2014). Prevalence, potential risk factors for development and symptoms related to the presence of uterine niches following Cesarean section: Systematic review. Ultrasound Obstet. Gynecol..

[3] Nezhat C., Falik R., Li A. (2017). Surgical management of niche, isthmocele, uteroperitoneal fistula, or cesarean scar defect: A critical rebirth in the medical literature. Fertil. Steril..

[4] Jacobson M.T., Osias J., Velasco A., Charles R., Nezhat C. (2003). Laparoscopic repair of a uteroperitoneal fistula. JSLS.

[5] Mashiach R., Burke Y.Z. (2021). Optimal Isthmocele Management: Hysteroscopic, Laparoscopic, or Combination. J. Minim. Invasive Gynecol..

[6] Di Spiezio Sardo A., Saccone G., McCurdy R., Bujold E., Bifulco G., Berghella V. (2017). Risk of Cesarean scar defect following single- vs. double-layer uterine closure: Systematic review and meta-analysis of randomized controlled trials. Ultrasound Obstet. Gynecol..

[7] Marchand G.J., Masoud A., King A., Ruther S., Brazil G., Ulibarri H., Parise J., Arroyo A., Coriell C., Goetz S. (2021). Effect of single- and double-layer cesarean section closure on residual myometrial thickness and isthmocele—A systematic review and meta-analysis. Turk J. Obstet. Gynecol..

[8] de Luget C.D., Becchis E., Fernandez H., Donnez O., Quarello E. (2021). Can uterine niche be prevented?. J. Gynecol. Obstet. Hum. Reprod..

[9] Tanos V., Toney Z.A. (2019). Uterine scar rupture—Prediction, prevention, diagnosis, and management. Best Pract. Res. Clin. Obstet. Gynaecol..

[10] Di Spiezio Sardo A., Zizolfi B., Calagna G., Giampaolino P., Paolella F., Bifulco G. (2018). Hysteroscopic Isthmoplasty: Step-by-Step Technique. J. Minim. Invasive Gynecol..

[11] Karampelas S., Salem Wehbe G., de Landsheere L., Badr D.A., Tebache L., Nisolle M. (2021). Laparoscopic Isthmocele Repair: Efficacy and Benefits before and after Subsequent Cesarean Section. J. Clin. Med..

[12] AbdullGaffar B., Almulla A. (2021). A Histopathologic Approach to Uterine Niche: What to Expect and to Report in Hysteroscopy-Resected Isthmocele Specimens. Int. J. Surg. Pathol..

[13] Karpathiou G., Chauleur C., Dridi M., Baillard P., Corsini T., Dumollard J.M., Peoc’H M. (2020). Histologic Findings of Uterine Niches. Am. J. Clin. Pathol..

[14] Birch Petersen K., Hoffmann E., Rifbjerg Larsen C., Svarre Nielsen H. (2016). Cesarean scar pregnancy: A systematic review of treatment studies. Fertil. Steril..

[15] Maheux-Lacroix S., Li F., Bujold E., Nesbitt-Hawes E., Deans R., Abbott J. (2017). Cesarean Scar Pregnancies: A Systematic Review of Treatment Options. J. Minim. Invasive Gynecol..

[16] Tan K.L., Chen Y.M., Zeng W., Meng Y., Jiang L. (2022). Local Methotrexate Injection Followed by Dilation and Curettage for Cesarean Scar Pregnancy: A Prospective Non randomized Study. Front. Med..

[17] Xiang J., Cao Y., Zhou L., Yang H., Wu S., Li L. (2022). Evaluation of the necessity of laparoscopic repair of a uterine scar defect for cesarean scar pregnancy. J. Int. Med. Res..

[18] Morlando M., Buca D., Timor-Tritsch I., Cali G., Palacios-Jaraquemada J., Monteagudo A., Khalil A., Cennamo C., La Manna V., Liberati M. (2020). Reproductive outcome after cesarean scar pregnancy: A systematic review and meta-analysis. Acta Obstet. Gynecol. Scand..

